# Erythema and hyperpigmentation delineation for cutaneous chronic graft-versus-host disease using hyperspectral reconstruction

**DOI:** 10.1364/BOE.610164

**Published:** 2026-09-02

**Authors:** Mayaank Pillai, Ruijing E. Han, Inga Saknite, Eric Tkaczyk, Audrey K. Bowden

**Affiliations:** 1 Vanderbilt University, Department of Computer Science, Nashville, TN, USA; 2 Vanderbilt University, Department of Biomedical Engineering, Nashville, TN, USA; 3 University of Latvia, Riga, Latvia; 4 Vanderbilt University Medical Center, Nashville, TN, USA; 5 Dermatology Service and Research Service, Department of Veterans Affairs, Tennessee Valley Healthcare System, Nashville, TN, USA

## Abstract

Erythema body surface area is a key indicator of cutaneous chronic graft-versus-host disease (cGVHD) status, but clinician estimation is unreliable and confounded by hyperpigmentation. Existing automated methods either fit predictive algorithms to subjective human annotations or require costly hyperspectral imagers. To our knowledge, we present the first annotation-free method to delineate both erythema and hyperpigmentation from standard RGB images. Our pipeline (i) reconstructs hyperspectral images from RGB input and (ii) unmixes these into hemoglobin and melanin abundance maps, which predict erythema and hyperpigmentation, respectively. Trained only on synthetic images, the method achieved a median binary classification accuracy above 0.70 across both conditions and both synthetic images and clinical photographs, demonstrating generalizability to the clinical environment.

## 1. Introduction

Chronic graft-versus-host disease (cGVHD), a multisystem disorder in which immune cells from a transplant donor (the graft) mount a chronic attack against the recipient's (host) tissues, is the leading cause of long-term morbidity and non-relapse mortality following allogeneic hematopoietic cell transplantation [1].

The skin is affected in 60-80% of cGVHD cases, and it plays a central role in evaluating disease progression and treatment efficacy because it is highly accessible for repeated examination [2]. A key manifestation of cutaneous cGVHD is erythema. Erythema includes several types of inflammatory cutaneous involvement, which are all characterized by increased cutaneous blood volume, and thus, increased blood-related chromophore (i.e., hemoglobin) concentration in the skin [1].

Change in erythema body surface area (BSA) is a key indicator of cGVHD status: even a 10% increase in erythema BSA confers significantly greater risk of death [1], and decreases in erythema BSA are also associated with improved survival. Thus, regular assessment of erythema BSA provides key information about disease progression and helps inform cGVHD treatment decisions. However, the clinical utility of erythema BSA is constrained by inter-rater disagreement in bedside clinician estimation, which is the current standard of care [3,4]. This approach involves the clinician visually delineating, or “segmenting”, the skin into erythematous and non-erythematous regions to estimate BSA. In this evaluation setting, the minimum detectable erythematous change required to exceed human rater measurement error is 19-22% BSA [3], suggesting that smaller changes in BSA—which are clinically relevant for monitoring cGVHD—may be indistinguishable from measurement noise when relying on clinician assessment alone.

Hyperpigmentation, reflecting increased cutaneous melanin, can further confound erythema BSA estimation, because pigmentary variation obscures the redness associated with inflammation [5]. Hyperpigmentation can be present prior to the development of erythema, or can reflect a post-inflammatory change [5]. In the latter case, hyperpigmentation can overlap with active erythema and may remain after erythema subsides [5]. Thus, while monitoring the extent of erythema in hyperpigmented regions is more difficult, it is also critical: clinicians must assess whether any active inflammation remains under the pigmentation, or if it has fully subsided. Together, these effects exacerbate the unreliability of visual assessment for monitoring cGVHD progression.

Various methods have been explored for automated segmentation of cutaneous cGVHD to enable consistent and reproducible erythema BSA estimation. McNeil et al. developed deep learning models to delineate erythema in cGVHD-affected skin using 2D or 3D images captured by standardized photography setups [6,7]. However, these approaches rely on fitting a model to human-drawn annotations, resulting in a model that is trained to reproduce a set of segmentations that may vary significantly by rater and patient skin tone. Thus, even if the model has high accuracy on a particular erythema label set, the learned delineations still reflect the annotation conventions of specific raters, rather than a reproducible measure of erythema. As a result, this approach can result in erythema BSA estimations with variability within the range reported for visual clinician inspection [7], rather than reducing inter-rater disagreement.

Liu et al. aimed to make model outputs more reliable by supervising training with more trustworthy annotations. Rather than asking a rater to segment an entire erythematous region, they restricted human annotations to sparse, high-confidence regions that were either definitely affected (‘Do Not Miss’, DNM) or definitely unaffected (‘Do Not Include’, DNI) by erythema [8]. Constraining supervision to these confident regions yields more reliable labels than fitting a model to unconstrained, whole-region annotations, where the rater attempts to segment the entire erythematous region. While this confident-annotation strategy improves label reliability, deploying it in practice introduces new challenges for clinical care. The presented approach also relies on a specialized camera ( $ 13,000 as tested) and pre-cutaneous-cGVHD baseline images to estimate erythematous regions, which may not be available in routine care. More critically, the DNI and DNM annotations still show inter-rater disagreement, and thus, this method also produces erythema segmentations that may reflect subjective rater preferences rather than underlying biology.

Existing machine learning frameworks for erythema BSA estimation—using human annotations of erythema to supervise model training—are particularly ill-suited for patients with hyperpigmentation. Because hyperpigmentation can mask underlying erythema [9], the annotations associated with these samples may be particularly unreliable, resulting in less accurate erythema BSA estimation.

Hyperspectral images (HSIs) contain more channels than traditional three-channel RGB images, which provide a detailed spectral characterization of the imaged region. HSIs of cGVHD-affected tissues have been shown to enable the automated segmentation of erythematous and hyperpigmented regions by estimating the abundance of hemoglobin and melanin, respectively [10], visualized as an abundance map. This approach does not fit a predictive algorithm to human annotations: it instead delineates erythema and hyperpigmentation using the spectral absorption properties of the chromophores captured in the physical images. Annotations are therefore reserved for evaluation—quantifying how closely the chromophore-derived delineations agree with an expert-approved assessment of erythema and hyperpigmentation—rather than serving as training targets. However, it relies on a specialized imager that is expensive (> $ 10,000 for commercial systems) and may be too bulky for a busy clinic environment [10].

In an effort to avoid expensive hyperspectral imagers, RGB-to-hyperspectral reconstruction models have been widely used to recover the dense spectral information typically found in HSIs, from RGB images [11], including for estimating skin hemodynamics [12]. For example, the Multi-stage Spectral-wise Transformer (MST++) architecture has shown state-of-the-art (SOTA) performance in spectral recovery competitions [13], and has been used to reconstruct hyperspectral data from RGB skin images [14]. Thus, we developed a sequential pipeline that first (i) uses spectral recovery to estimate HSIs from RGB input and then (ii) generates hemoglobin and melanin abundance maps by unmixing these predicted HSIs.

To our knowledge, this work presents the first method to use 2D RGB images to delineate both erythema and hyperpigmentation for cutaneous cGVHD. Importantly, the results produced here are physics-informed (via chromophore estimation) and do not rely on human annotations, which are prone to errors due to subjective assessment. By providing delineations of both erythema and hyperpigmentation, our methods aim to reduce the confounding effect of pigmentary changes on erythema estimation by allowing for easier interpretation of regions containing both. The ability to perform chromophore-directed erythema delineation on standard photographs makes our approach more accessible and practical for routine monitoring than one relying on a hyperspectral imager.

## 2. Methods

### 2.1. Overview

The primary goal of our work is to reliably predict the extent of erythema from RGB images, even with the presence of hyperpigmentation. Our strategy is to first convert RGB images into hyperspectral datasets, from which we use spectral estimation to determine the presence of chromophores associated with both conditions. RGB-to-hyperspectral reconstruction models estimate per-pixel spectra, mapping an RGB image *x* of shape (3,H,W) to a reconstructed HSI $\hat{H}$ of shape (C,H,W) , where C is the number of spectral channels, called bands (commonly 10-120) [11].

HSIs can then be “unmixed” into spectra associated with individual absorbers (i.e., endmember spectra) with corresponding abundance maps, which describe each endmember’s relative per-pixel contribution. Thus, HSIs, including those capturing the skin, are often modeled as a linear mixture of endmember spectra and their corresponding abundance maps [15]. Consider abundance maps *A* of shape (N,H,W) and endmember spectra *E* of shape (N,C) . A given length- C spectrum $h_{i}$ at pixel *i* of HSI *H* can be modeled as

(1) $$h_{i}=E^{⊤}a_{i},$$

where $a_{i}$ are the corresponding abundances in *A*. Equivalently, at each HSI pixel, the C -band spectrum is a linear combination of N endmember spectra and N abundances. To model cutaneous HSIs, these endmembers can be selected as the spectra of known absorbers in the skin (e.g., hemoglobin and melanin). Our goal, therefore, is to unmix each reconstructed HSI $\hat{H}$ , with the selected endmembers modeling the absorbance of hemoglobin, melanin, or an offset that captures baseline imaging effects (e.g., scattering) and other cutaneous chromophores. The resulting abundance maps then approximate the relative contributions of these desired chromophores at each image pixel.

At the highest level, our pipeline (Fig. 1) thus consists of two stages: (i) RGB → HSI reconstruction and (ii) HSI unmixing to yield the abundance maps. In what follows, we first describe the data underlying our work: our source dataset (Sec. 2.2) and the procedures used to prepare the paired RGB and HSI data it requires (Sec. 2.3). We then detail how we implemented the two pipeline stages (Sec. 2.4). Finally, we discuss how we combined these components in our experiments and how we evaluated the resulting HSI reconstructions and abundance maps (Sec. 2.5).

**Fig. 1.**
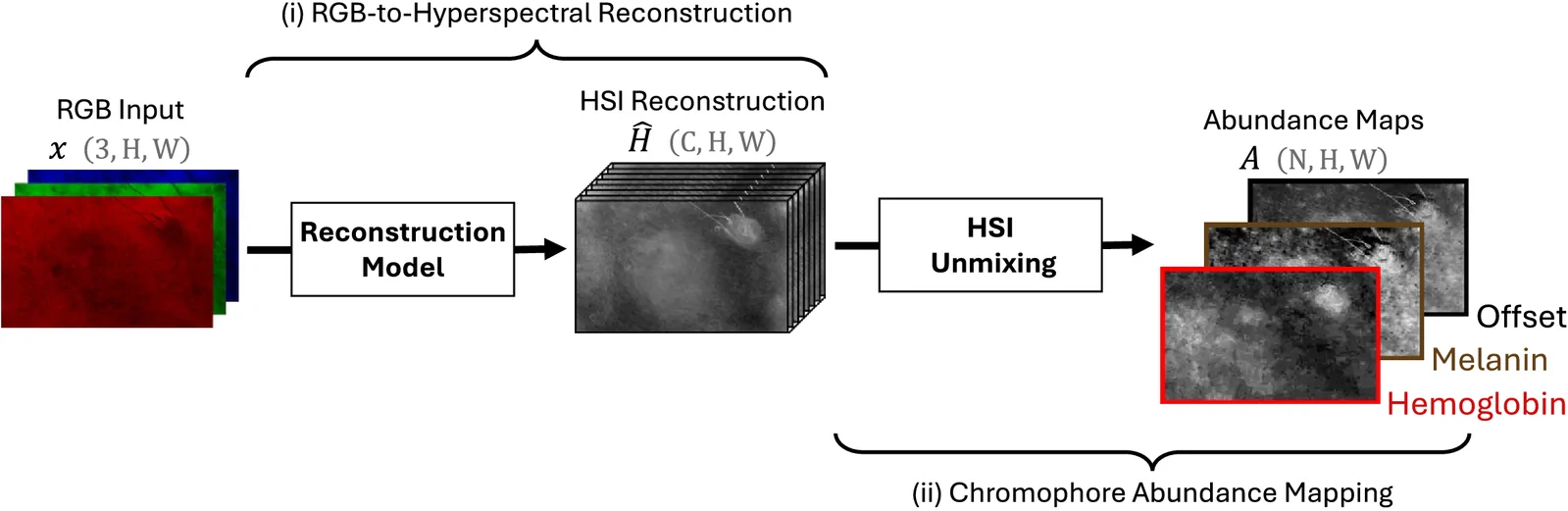
An overview of our two-stage hemoglobin and melanin abundance mapping pipeline on RGB images. In this work, RGB inputs represent digital camera images or synthetic RGB images generated from hyperspectral data. Hemoglobin maps obtained from reconstructed HSIs are used to predict erythema, and similarly derived melanin maps are used to predict hyperpigmentation.

### 2.2. Data source

For this work, we adopted a cutaneous cGVHD HSI dataset previously collected by Saknite et al. [10]. For this study, patients with cutaneous cGVHD were enrolled without restriction on age, sex, or race, and representative 2.5 × 3.5 cm areas of affected skin were imaged with a handheld prototype hyperspectral imager (Revenio Group, Finland) capturing 120 bands spanning 450–850 nm. Acquisition conditions were held fixed across the hyperspectral collections: illumination was provided by a halogen-based (150 W EKE quartz halogen) fiber-optic illuminator (Dolan-Jenner DC-950) and a ring light, and a black 3D-printed spacer enclosed the lens, ring light, and imaged area, maintaining a constant ∼4 cm lens-to-skin distance and excluding ambient light. A white reference, which was a sheet of white paper supplied by the manufacturer rather than a calibrated reflectance standard, was imaged in addition to each cutaneous measurement. The resulting dataset comprised 51 samples from 15 patients with cGVHD, where each sample consists of a cutaneous hyperspectral measurement and its paired white-reference hyperspectral measurement. For seven of these patients, RGB images had also been collected with a digital camera, producing 18 clinical photographs; each photograph had been cropped and manually aligned (but not perfectly registered) to the hyperspectral measurement of its corresponding sample.

The dataset also contained high-confidence expert-approved annotations of erythema and hyperpigmentation. For a given condition (erythema or hyperpigmentation) present in a sample, a trained rater delineated “definitely affected” (do not miss, DNM) and “definitely unaffected” (do not include, DNI) regions; we refer to this paired DNM/DNI delineation for a single condition as a label set. A sample therefore had one label set for each condition present in it (at most two). Annotation was performed on the clinical photograph when available and otherwise on the RGB rendering of the HSI measurement output by the imager software. Final annotations were approved by a board-certified dermatologist (ET).

Of the 51 samples, 48 exhibited erythema and 27 exhibited hyperpigmentation (24 with erythema only, 3 with hyperpigmentation only, and 24 with both conditions), yielding 48 erythema label sets and 27 hyperpigmentation label sets—75 label sets in total. Table 1 summarizes the dataset.

**Table 1.** An overview of the dataset collected by Saknite et al. [10]*^a^*

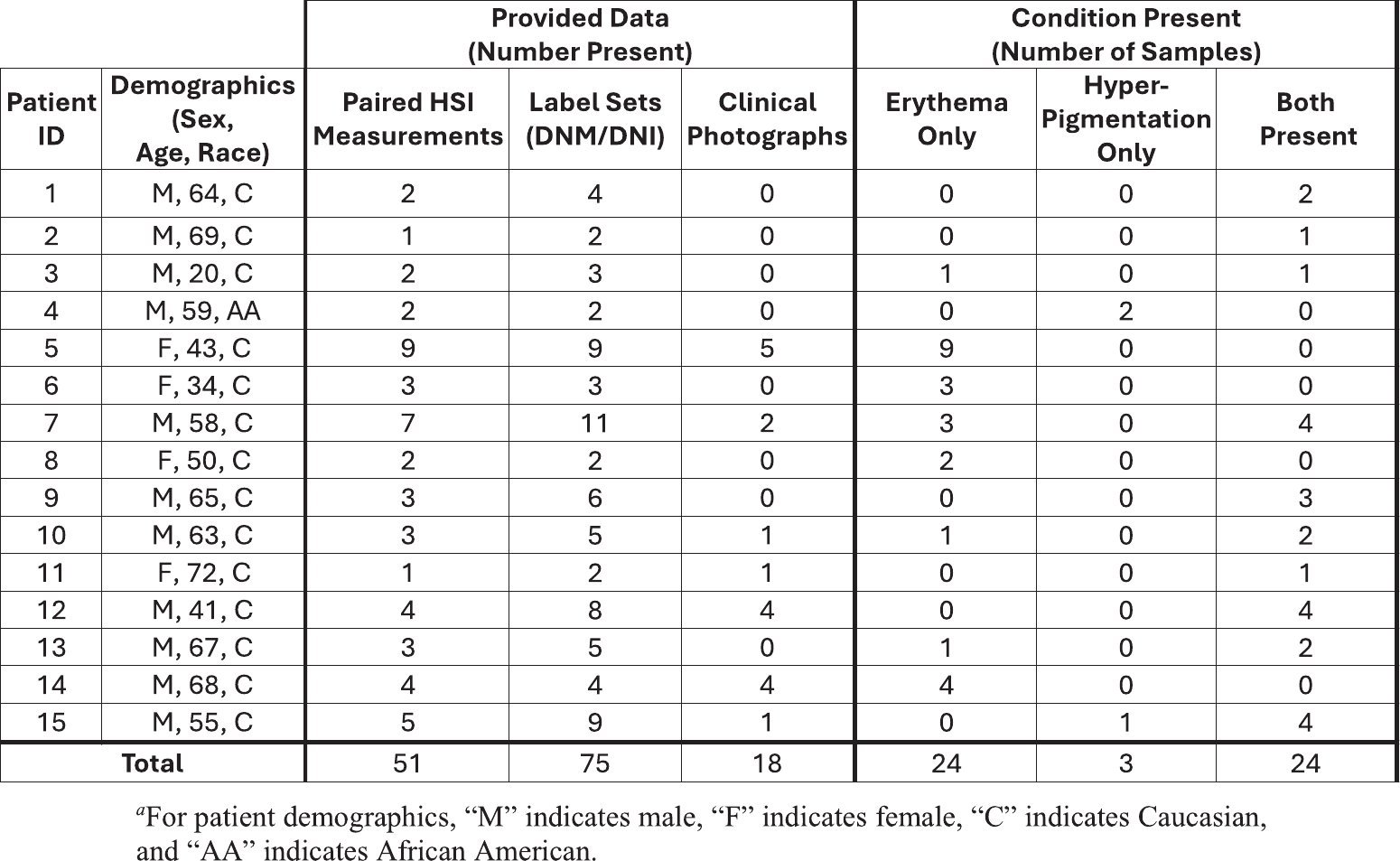

### 2.3. Dataset generation

As noted in Fig. 1, the first stage of our pipeline reconstructs an HSI from an RGB image. Training and evaluating this stage thus required paired data for each sample: an RGB image to serve as the input and a corresponding ground truth HSI to serve as the reconstruction target. We first processed the cutaneous HSI and white-reference measurements (Sec. 2.3.1) to derive one ground truth HSI per sample. For the RGB inputs, however, our source data were insufficient: they did not contain clinical photographs for all patients, and we found the manufacturer-produced RGB renderings of insufficient quality to train a successful pipeline. To generate a reliable RGB image for each sample, our strategy aligned with the standard in spectral recovery work [11]: that is, we rendered each sample's ground truth HSI into a realistic RGB image, hereafter referred to as a synthetic image to distinguish it from a native clinical photograph (Sec. 2.3.2).

#### 2.3.1. HSI ground truth generation

*1. HSI Measurement Processing:* For each sample, we used manufacturer-provided (Revenio Group) code to convert the cutaneous and white-reference measurements into HSIs in sensor units. We then divided the cutaneous HSI by the white-reference HSI to obtain a normalized HSI in reflectance units and converted it to absorbance units by clamping reflectance to [0.01, 1] and taking the negative base-ten logarithm of each element [15]. We then discarded the channels corresponding to wavelengths above 700 nm, the spectral response limit of common RGB cameras [17], so they were excluded from all subsequent processing. The resulting HSI contained 86 bands spanning [450,700] nm.

*2. Inter-band Registration:* Because each hyperspectral collection took several seconds, movement of the handheld imager caused misalignment of consecutively captured HSI bands. For each HSI, we used SimpleITK and Elastix to register each band to a reference band [16]: we chose the band at the midpoint of the acquisition to be the reference band. Each band was aligned to the reference using a multi-resolution B-spline free-form deformation optimized with Mattes mutual information. Final resampling used nearest-neighbor interpolation.

*3. Spectral Denoising:* We further processed each sample HSI by using Savitzky-Golay spectral filtering to mitigate noise and band-wide intensity shifts. We selected a polynomial order of three and a window length of seven bands.

*4. Spectral Downsampling:* To reduce spectral redundancy, we downsampled each HSI by selecting every other band from the 86 available, producing a 43-band HSI. This was chosen to be comparable to the band counts used in standard spectral recovery work (e.g., the 31 bands of the ARAD1 K benchmark [11]), and we did not attempt to optimize this number. This yielded 51 ground truth HSIs (one per sample), which served as the ground truth HSIs for the remainder of the pipeline.

#### 2.3.2. Synthetic image generation

Our procedure to generate a synthetic RGB image centers on a single differentiable rendering model that maps an input ground truth HSI spectrum to an output RGB pixel. First, we describe how we parameterized the rendering model to enable this spectrum-to-pixel mapping (Sec. 2.3.2.1). Second, we detail how we preprocessed our data and trained the rendering model such that its output resembled clinic imaging conditions (Sec. 2.3.2.2). Third, we discuss how we applied this trained model to ground truth HSIs to predict synthetic images (Sec. 2.3.2.3).

##### 2.3.2.1. Rendering model parameterization

The synthetic image generation process required a mapping from HSI spectra to RGB pixels. At each pixel *i*, the hyperspectral input was represented as a reflectance vector

(2) $$h_{i}={\left[h_{i,1},\ldots ,h_{i,\;B}\right]}^{⊤}\in {\left[0,1\right]}^{B},$$

sampled at wavelengths $\lambda ={\left[\lambda_{1},\;\ldots ,\;\lambda_{B}\right]}^{⊤}$ . For our ground truth HSIs, we had B=43 bands over the wavelength range [450nm,700nm] . The RGB output was a linear vector $x={\left[x_{R},x_{G},\;x_{B}\right]}^{⊤}$ .

In RGB rendering work, the linear response of a given channel *c* for an RGB pixel is often represented via a discretized Lambertian reflectance model:

(3) $$x_{i,c}^{\mathrm{raw}}=\overset{B}{\underset{b=1}{\sum }}h_{i,b}E_{b}S_{c,b}\Delta \lambda_{b},$$

where $E_{b}$ is the illuminant spectral power distribution (SPD) evaluated at wavelength $\lambda_{b}$ , $S_{c,b}$ is the spectral sensitivity of channel *c* at $\lambda_{b}$ , and $\Delta \lambda_{b}$ is the trapezoidal integration weight for band *b*. This equation captures the interaction between target surface reflectance, scene illumination, and imager-specific spectral response to estimate RGB pixel-values. Representing a full linear RGB pixel requires three channel responses, one for each c∈{R,G,B} .

The spectral sensitivities $S_{c,b}$ and illuminant values $E_{b}$ are typically selected to model desired cameras or illumination conditions. However, our dataset contained 18 true clinical photographs, each manually aligned with an HSI. Instead of hand-selecting these curves, we chose to optimize them such that the produced synthetic images resembled their corresponding photographs and thus were aligned with clinic imaging conditions. To enable this, we parameterized Eq. (3) with common approximations to produce a differentiable rendering model.

**Parameterizing** $S_{c,b}$ **:** In spectral sensitivity estimation work, these spectral sensitivities are often modeled as a mixture of Gaussian basis functions to characterize camera-specific responses [17]. We chose to parameterize the camera spectral sensitivity for each channel as a mixture of three Gaussian components. For each channel c∈{R,G,B} and component k∈{1,2,3} , we learned the parameters

(4) $${\tilde{a}}_{c,k},{\tilde{\mu }}_{c,k},{\tilde{\sigma }}_{c,k},$$

which were constrained to represent physically possible ranges

(5) $$a_{c,k}=reLU({\tilde{a}}_{c,k})+\varepsilon ,$$

(6) $$\mu_{c,k}=\lambda_{\text{min}}+\sigma ({\tilde{\mu }}_{c,k})(\lambda_{\text{max}}-\lambda_{\text{min}}),$$

(7) $$\sigma_{c,k}=reLU({\tilde{\sigma }}_{c,k})+\varepsilon ,$$

where $\sigma (x)=\frac{1}{1+e^{-x}}$ is the standard logistic sigmoid function and $\varepsilon =10^{-8}$ is used for numerical stability. The resulting spectral sensitivity vector was

(8) $$S_{c,b}=\overset{3}{\underset{k=1}{\sum }}a_{c,k}\exp\left(-\frac{1}{2}{\left(\frac{\lambda_{b}-\mu_{c,k}}{\sigma_{c,k}}\right)}^{2}\right),$$

for b∈{1,…,B} , where exp is the standard exponential function.

**Parameterizing** $E_{b}$ **:** Illumination conditions can be approximated by a Planckian radiator, enabling the illuminant SPD to be parameterized by a learnable color temperature *T*. To ensure positive temperatures we learned a training parameter $\ell_{T}$ , and defined

(9) $$T=(T_{\text{max}}-T_{\text{min}})\;\sigma (\ell_{T})+T_{\text{min}},$$

where $T_{\text{max}}=7,000$ and $T_{\text{min}}=4,000$ were selected to constrain the illuminant to a moderate white-light range, covering daylight-like color temperatures commonly used for imaging (e.g., CIE D65 and D50 illuminants) [18]. The SPD was then

(10) $$B_{b}(T)=\;\frac{2\;h\;c^{2}}{{\left(\lambda^{\left(m\right)}\right)}^{5}}\;\cdot \;\frac{1}{\exp\left(\frac{h\;c}{\lambda^{\left(m\right)}k_{B}T}\right)-1},\;\;\;$$

where $\lambda^{\left(m\right)}=\lambda \times 10^{-9}$ converts wavelength in nm to meters, *h* is the Planck constant, $k_{B}$ is the Boltzmann constant, and *c* is the speed of light in a vacuum [19]. For reasonable scaling with the other rendering model components, our final SPD was normalized by its maximum value in our wavelength range

(11) $$E_{b}(T)=\frac{B_{b}(T)}{\underset{\lambda \in [\lambda_{\min},\lambda_{\max}]}{\max}B_{b}(T)+\varepsilon }\;,$$

with $\varepsilon =10^{-8}$ for numerical stability.

The raw linear RGB response for a spectrum $h_{i}$ was then computed as

(12) $$x_{i}^{\mathrm{raw}}=\left[\begin{array}{l} \overset{B}{\underset{b=1}{\sum }}h_{i,\;b}E_{b}S_{R,b}\Delta \lambda_{b}\\ \overset{B}{\underset{b=1}{\sum }}h_{i,\;b}E_{b}S_{G,b}\Delta \lambda_{b}\\ \overset{B}{\underset{b=1}{\sum }}h_{i,\;b}E_{b}S_{B,b}\Delta \lambda_{b} \end{array}\right],$$

by substituting Eqs. (8) and (11) into Eq. (3).

While this Lambertian reflectance rendering model provides a physics-guided approach to generating linear RGB estimations of HSI spectra, additional color space transformations are often required to adjust the rendered images [20]. Our HSIs did not have any reflectance values corresponding to wavelengths below 450 nm, which resulted in an inadequate contribution to the blue channel. Additionally, white reference data were collected using a sheet of white paper, rather than a calibrated standard, which introduced color distortion in the resulting linear RGB images. To correct these factors, we applied a learned 3×3 color correction matrix *C* to $x_{i}^{\mathrm{raw}}$ and clamped the pixel to non-negative values,

(13) $$x_{i}=\text{clamp}(Cx_{i}^{raw},0,\infty ),\;$$

producing our final linear RGB pixel estimation.

##### 2.3.2.2. Data preprocessing and rendering model training

To train our rendering model to produce reliable synthetic images, we needed to optimize its learnable parameters: the per-channel spectral sensitivity Gaussian variables $\left({\tilde{a}}_{c,k},{\tilde{\mu }}_{c,k},{\tilde{\sigma }}_{c,k}\right)$ , the illuminant color temperature *T*, and the color correction matrix *C*. To enable this, we implemented our rendering model in PyTorch. We first performed preprocessing on our clinical photographs and ground truth HSIs. Then, we trained the rendering model using ground truth HSI and clinical photograph pairs. Figure 2 provides an overview of our data preprocessing steps and the rendering model training loop.

**Fig. 2.**
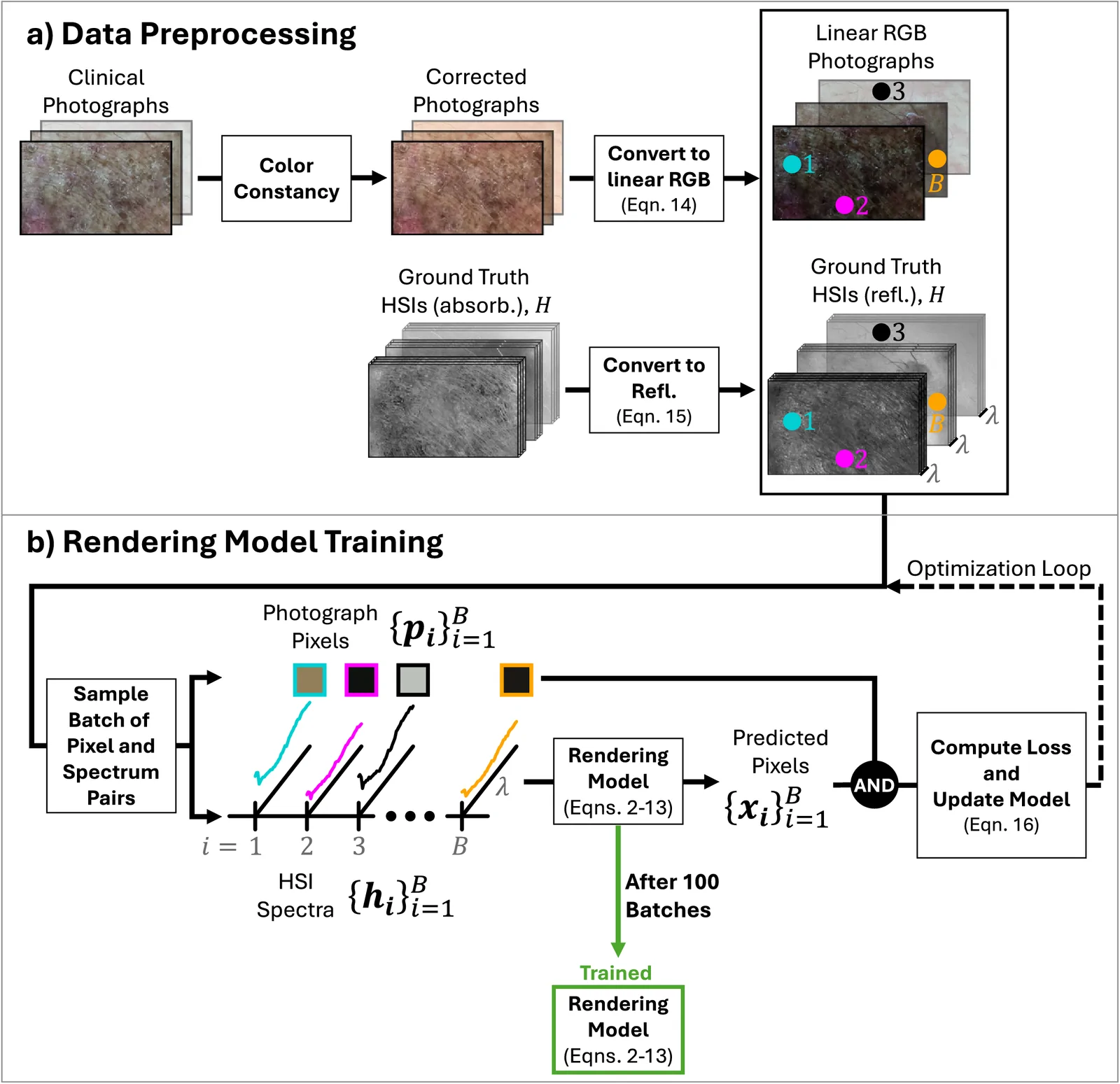
An overview of our preprocessing and rendering model training steps for pairs of ground truth HSIs and clinical photographs. After 100 batches, training is halted, yielding the trained rendering model.

**Data Preprocessing:** Prior to training, we applied color constancy transforms to each clinical photograph to mitigate the effects of variable lighting and posing across our targets. We specifically used the Shades-of-Gray algorithm (using Minkowski p-norm, p=6 ) [21] and von Kries diagonal correction [22]. Digital cameras store images in the sRGB color space, where pixel values are gamma-encoded and therefore are a nonlinear function of scene radiance. Our rendering model provided linear RGB predictions that were directly proportional to radiance. To make the photographic targets consistent with this linear model output, we reversed the camera-applied gamma encoding. For each sRGB pixel s in each photograph, we computed the linear RGB pixel with

(14) $$p_{c}=\{\begin{array}{ll} \frac{s_{c}}{12.92}, & s_{c}\leq 0.04045\;\\ {\left(\frac{s_{c}+0.055}{1.055}\right)}^{2.4}, & s_{c}>0.04045\; \end{array}\;,$$

for each channel c∈{R,G,B} , which followed the standard sRGB conversion formulation [23].

To preprocess our ground truth HSIs, we converted them from absorbance to reflectance units. For each spectrum $a_{i}$ in each ground truth HSI, we computed

(15) $$h_{i,b}=10^{-a_{i,\;b}},$$

at each wavelength band *b*. The resulting ground truth HSI spectrum *h* and photograph pixel *p* pairs would be used to train the rendering model.

**Rendering Model Training:** We trained the model for 100 batches using the Adam optimizer ( $\beta_{1}=0.9$ , $\beta_{2}=0.99$ , learning rate $10^{-3}$ ) and by randomly sampling batches of 1,024 ground truth HSI spectrum and clinical photograph pixel pairs. The output of our rendering model was a linear RGB vector $x_{i}$ with arbitrary scale. Our goal in training the rendering model was to optimize a spectrum-to-RGB conversion that was aligned with the color distributions seen in the clinical imaging environment. However, the clinical photographs contained factors that affect RGB pixel intensity but are not determined solely by the tissue reflectance spectrum, such as illumination and camera posing and shadows. These factors are not mirrored in the ground truth HSIs, which were collected with a standardized setup. If the renderer were trained with an intensity-matching RGB loss (e.g., a Mean Squared Error loss), the model could be driven to explain these nuisance brightness differences rather than the spectral-to-color relationship. To prevent this, we used an angle alignment loss between normalized predicted RGB pixels x and ground truth clinical photograph pixels p . For each batch, $\{\left(x_{i},p_{i}\right){\}}_{i=1}^{B}$ with B=1024 , the training objective was

(16) $$L=\frac{1}{B}\overset{B}{\underset{i=1}{\sum }}\left[1-\frac{x_{i}^{⊤}p_{i}}{\left({x_{i}}_{2}+\varepsilon )({p_{i}}_{2}+\varepsilon \right)}\right],$$

$\varepsilon =10^{-8}$ for numerical stability.

##### 2.3.2.3. Synthetic image prediction

Once training was completed, we could use the optimized rendering model to generate synthetic images from ground truth HSIs. Figure 3 provides an overview of this process for predicting synthetic images.

**Fig. 3.**
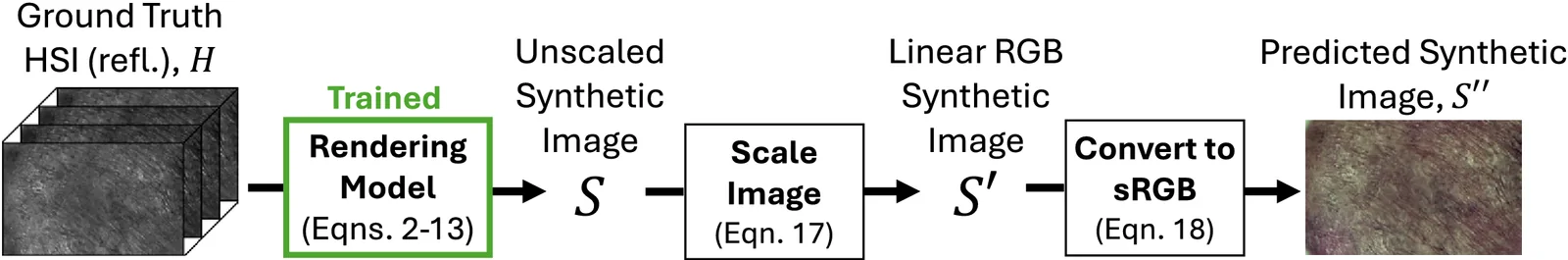
The steps for predicting synthetic images from our trained rendering model. The unscaled synthetic image *S* is predicted per-pixel from the spectra of the ground truth HSI.

We started with a given ground truth HSI in reflectance units. Then, we predicted the linear RGB pixel for each spectrum in the HSI, producing a synthetic linear RGB image $S\in R^{3\times H\times W}$ . However, the rendering model computes linear RGB pixels with arbitrary scale, with our training objective only optimizing the predicted hues but not pixel intensities. Thus, we applied a per-image scaling factor to each synthetic image to provide an image in the standard [0,1] pixel-value range. Let α be the 0.995 quantile value in the predicted image *S*, selected to prevent extreme predicted values from affecting the global scaling. The scaled synthetic image was

(17) $$S^{\prime }=\text{clamp}\left(\frac{1}{\alpha }S,\;\;\;0,\;\;\;1\right),$$

with the clamp function used to clip the image values to the desired pixel-value range. Finally, the synthetic image was converted to a gamma-compressed sRGB using the standard function [23]. For each pixel $s^{\prime }$ and channel c∈{R,G,B} , we applied

(18) $$s_{c}^{\prime \prime }=\{\begin{array}{ll} 12.92s_{c}^{\prime }, & s_{c}^{\prime }\leq 0.0031308\\ 1.055{\left(s_{c}^{\prime }\right)}^{\frac{1}{2.4}}-0.055, & s_{c}^{\prime }\;>0.0031308\; \end{array}\;,$$

to produce our final rendered synthetic image $S^{\prime \prime }$ .

### 2.4. HSI reconstruction and unmixing pipeline

As indicated by Fig. 1, our proposed pipeline had two stages: RGB-to-hyperspectral reconstruction performed by a reconstruction model, and chromophore abundance mapping performed by HSI unmixing. Thus far, we have discussed our data source (Sec. 2.2) and dataset generation methods (Sec. 2.3) required for training and evaluating this pipeline. Now, we detail our architecture implementation and training methods for the HSI reconstruction model and HSI unmixing.

#### 2.4.1. Reconstruction model

**Architecture:** We adopted the MST++ spectral recovery architecture for the task of HSI prediction from an RGB input. This architecture uses a cascade of Single-stage Spectral-wise Transformer (SST) blocks, each of which is composed of an encoder-decoder arrangement of Spectral-wise Attention Blocks (SABs) [13]. We made two configuration changes to the reference implementation provided by Cai et al. First, we used only one SST block to mitigate overfitting on our limited dataset. Second, we adjusted the number of predicted spectral channels to C = 43, matching the channel count of our ground truth HSIs. We made no changes to the SST or SAB components, and thus refer to Cai et al. for implementation details [13].

**Training Details:** To train MST++, we used random 256×256 patches of each input synthetic image *x* and the corresponding ground truth HSI *H*. We used a standard Mean Squared Error (MSE) optimization objective between the reconstructed and ground truth HSI patches, computed over all 43 reconstructed bands. We used the AdamW optimizer (­ $\beta_{1}=0.9$ , $\beta_{2}=0.99$ , initial learning rate $10^{-3}$ , weight decay $10^{-4}$ ), a batch size of six, and a ReduceLROnPlateau scheduler. The learning rate was halved if the validation loss plateaued for five epochs, and we stopped training once at least 30 epochs had passed and the validation loss stalled for ten epochs. The output of this process was a trained model that we could use to predict HSI reconstructions from input RGB images (e.g., held-out synthetic images or clinical photographs).

#### 2.4.2. HSI unmixing

For the second step of our proposed pipeline, we required an unmixing algorithm that could estimate hemoglobin and melanin abundance maps from predicted HSI reconstructions. A previously explored, baseline approach would be to perform per-spectrum least-squares regression on the reconstructed HSI using reference endmembers (e.g., the absorbance spectra of hemoglobin and melanin) [10]. This would be a direct implementation of the formulation seen in Eq. (1). However, this assumes that the reference endmembers are fixed and accurately represent the spectra in every reconstructed HSI, despite image-specific spectral variability being common [24]. To relax this assumption, for each reconstructed HSI, we first optimized an additive adjustment matrix *M* that augmented the reference endmembers $E_{\text{REF}}$ and then used these adjusted endmembers *E* for unmixing. Additionally, rather than using the baseline least squares regression, we used an updated unmixing regression method to produce more plausible abundance maps.

Figure 4 provides an overview of our abundance mapping method, including our procedure for optimizing the adjustment *M* to yield optimized endmembers (Fig. 4(a)), and our method for predicting the final abundance maps *A* (Fig. 4(b)). We first discuss how we selected the reference endmembers and how we computed adjusted endmembers. Then, we discuss how our unmixing regression method estimated chromophore abundances from reconstructed HSI spectra using these adjusted endmembers. Finally, we detail how we optimized *M* for each reconstructed HSI and produced our final abundance map predictions.

**Fig. 4.**
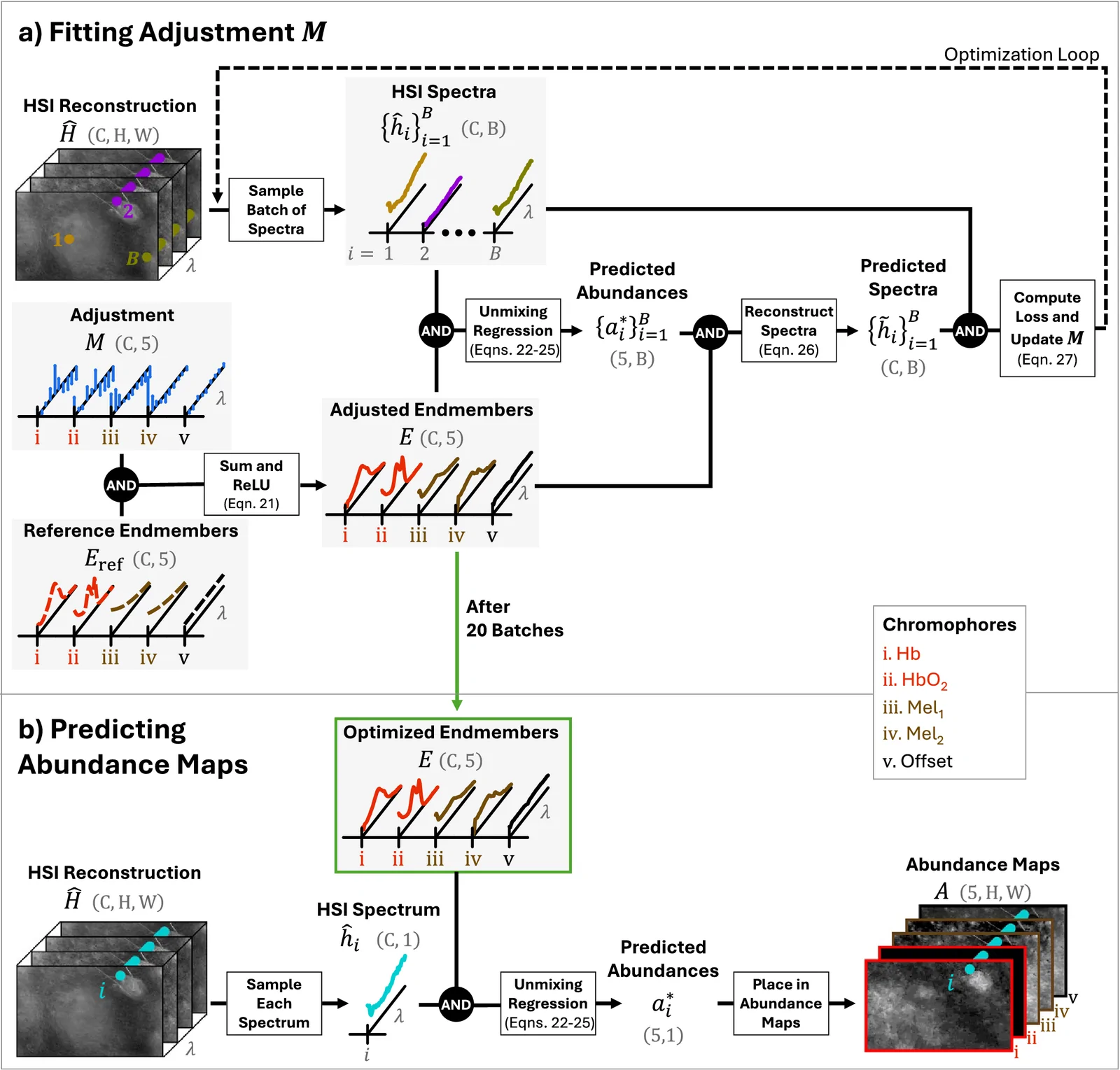
An overview of our HSI unmixing method. Adjusted endmembers *E* are computed as a sum of reference endmembers $E_{\text{ref}}$ and a learned adjustment *M*, with a reLU used for non-negativity. We perform unmixing regression on HSI reconstruction spectra using these adjusted endmembers to predict abundances $a_{i}^{*}$ . (a) We first fit the adjustment by sampling spectra from the HSI reconstruction $\hat{H}$ and optimizing *M*. (b) After 20 batches, we use the finalized adjustment *M* to produce optimized endmembers. We predict the abundance maps *A* via unmixing regression by sampling each spectrum in $\hat{H}$ and using these optimized endmembers.

**Reference Endmembers:** To unmix reconstructed HSIs into hemoglobin- and melanin-specific abundance maps, we first required absorbance spectra, which would serve as reference endmembers, for these chromophores. We constructed these spectra based on the band-center wavelengths for the channels in our reconstructed HSIs. We used the tabulated molar extinction coefficients measured for oxygenated and deoxygenated hemoglobin by Prahl [25] and linearly interpolated them to our target wavelengths. We then converted these extinction coefficients to absorbance spectra using

(19) $$E_{\text{REF},1}=\varepsilon_{\text{Hb}}\frac{150\;\text{g/l}}{65,000\;\text{g/mole}},\;E_{\text{REF},2}=\varepsilon_{\text{Hb}O_{2}}\frac{150\;\text{g/l}}{65,000\;\text{g/mole}},$$

where $\varepsilon_{\text{Hb}}$ and $\varepsilon_{\text{Hb}O_{2}}$ are the interpolated extinction coefficient spectra, 150 g/liter is the whole-blood hemoglobin concentration, and 65,000 g/mole is the hemoglobin molecular weight. Melanin was modeled using the skin melanosome approximation [26]

(20) $$\mu_{a}(\lambda )=1.70\times 10^{12}\lambda^{-3.48},$$

which provides the absorbance coefficient $\mu_{a}$ for each target wavelength λ corresponding to the bands in our reconstructed HSIs. This was converted to an absorbance spectrum by dividing by ln⁡10 . To better capture the variability in melanin-related spectral shape, we included a second melanin-specific reference endmember with a shallower power-law dependence on wavelength, proportional to $\lambda^{-2.48}$ . We refer to these melanin spectra as Mel_1_ and Mel_2_, respectively. A final constant, flat reference endmember was included to model broadband scattering and varying illumination, referred to as the offset endmember. These five spectra—corresponding to Hb, HbO_2_, Mel_1_, Mel_2_, and the offset—were used as the reference endmembers $E_{\text{REF}}$ of shape (C,5) for unmixing as seen in Fig. 4.

**Adjusted Endmembers** E **:** To make our unmixing approach more adaptable to spectral variability, we used an additive adjustment matrix *M* of shape (C,5) for each reconstructed HSI. Thus, our unmixing algorithm used the endmembers

(21) $$E=\text{reLU}(E_{\text{REF}}+M)+\varepsilon ,$$

where the reLU function is used to enforce non-negativity and $\varepsilon =10^{-8}$ is used for numerical stability. We first fit *M* for each reconstructed HSI, before using the adjusted endmembers *E* for unmixing.

**Unmixing Regression:** Let ${\hat{h}}_{i}$ be the length- C spectrum at pixel *i* in the reconstructed HSI $\hat{H}$ . Given the adjusted endmembers *E* from Eq. (21), the abundances $a_{i}^{*}$ for ${\hat{h}}_{i}$ were estimated by solving the non-negative ridge regression problem

(22) $$a_{i}^{*}=\arg\;\underset{a\geq 0}{\min}\frac{1}{2}{\left||{\hat{h}}_{i}-Ea|\right|}_{2}^{2}+\frac{\lambda_{r}}{2}a_{2}^{2},$$

where the ridge coefficient was selected as $\lambda_{r}=10^{-2}$ . To solve for non-negative $a_{i}^{*}$ , abundances were initialized as the standard unconstrained closed-form ridge solution [27]

(23) $$a_{i}={\left(E^{⊤}E+\lambda_{r}I\right)}^{-1}E^{⊤}{\hat{h}}_{i},$$

and then were projected onto the nonnegative orthant. The projected estimate was refined using 25 projected-gradient iterations,

(24) $$a_{i}^{\left(t+1\right)}=\text{clamp}[a_{i}^{\left(t\right)}-\eta ((E^{⊤}E+\lambda_{r}I)a^{\left(t\right)}-E^{⊤}{\hat{h}}_{i}),\;\;\;0,\;\;\;\infty ],$$

with η indicating step size and clamp indicating the nonnegative projection (i.e., setting each abundance estimate to minimum zero). We used a fixed step size

(25) $$\eta =\frac{1}{{\text{λ}}_{\text{max}}(E^{⊤}E+\lambda_{r}I)},$$

with ${\text{λ}}_{\text{max}}$ being a function that selects the largest eigenvalue, based on a common projected-gradient optimization approach [28,29]. We chose to use non-negative ridge regression rather than non-negative least squares (NNLS) because NNLS does not encode the physiological prior that skin spectra contain coexisting contributions from hemoglobin, melanin, and scatterers. For example, strongly erythematous tissue is still expected to contain melanin, and highly pigmented tissue is still expected to contain hemoglobin. The ridge penalty acts as a prior against sparse (i.e., one-endmember) unmixing of the reconstructed spectra, which we found results in more physiologically plausible abundances and discourages heavily disjoint hemoglobin and melanin maps.

**Fitting Adjustment Matrix** M **:** We implemented our HSI reconstruction unmixing approach in PyTorch, where *M* was a learnable parameter initialized to zero. For each reconstructed HSI $\hat{H}$ , we sampled batches of spectra $B=\{{\hat{h}}_{i}{\}}_{i=1}^{B}$ and solved for abundances $a_{i}^{*}$ via Eq. (22). The resulting reconstructed spectra (i.e., linear mixture of estimated abundances and endmembers) were computed as

(26) $${\tilde{h}}_{i}=Ea_{i}^{*},$$

for each ${\hat{h}}_{i}$ in the minibatch. The adjustment *M* was optimized using the objective function

(27) $$L_{B}=\frac{1}{BC}\overset{B}{\underset{i=1}{\sum }}{\left||{\tilde{h}}_{i}-{\hat{h}}_{i}|\right|}_{2}^{2}+\lambda_{2}{\left||M|\right|}_{F}^{2},\;$$

where weight $\lambda_{2}=10^{-3}$ was chosen to regularize the adjustment and prevent excessive drift from the reference endmembers. We used the Adam optimizer (­ $\beta_{1}=0.9$ , $\beta_{2}=0.99$ , learning rate $10^{-3}$ ), and trained for 20 batches of 5,000 randomly sampled spectra.

**Predicting Abundance Maps** A **:** After fitting *M* and thus obtaining the optimized endmembers *E*, we could produce abundance maps by solving for $a_{i}^{*}$ via Eq. (22) for each pixel index *i* in $\hat{H}$ . This produced five abundance maps, with two corresponding to hemoglobin (Hb and HbO_2_), two corresponding to melanin (Mel_1_ and Mel_2_), and one corresponding to the offset, as determined by the reference endmembers (Fig. 4). For subsequent analysis, we summed the Hb and HbO_2_ maps to produce a single hemoglobin estimation and similarly summed the two melanin maps to produce a single melanin estimation. We did not use the offset abundance map for erythema or hyperpigmentation delineation.

### 2.5. Experimental design and evaluation

Having defined our pipeline's architecture and training methods and our dataset generation procedure, we now describe the experiments used to train and evaluate the full pipeline on unseen data. We first detail our data splitting strategy and the per-split experimental procedure and then describe how we evaluated the resulting HSI reconstructions and chromophore abundance maps.

#### 2.5.1. Data splitting and experimental procedure

We organized our experiments around patient-wise data splits, performing an identical sequence of steps for each split. Because RGB-to-hyperspectral reconstruction requires supervised training, we needed to split our data into training, validation, and testing sets to enable evaluation on unseen data. We split the data by patient: all samples from a given patient were assigned exclusively to one of these sets. For each split, we selected one patient as the test set, a random different patient as the validation set, with the remaining patients forming the training set. This data splitting method yielded 15 distinct data splits (one per patient). We first generated our set of 51 ground truth HSIs, which would be shared across our 15 experiments (Sec. 2.3.1). Then, we performed five sequential experimental steps for each data split (Fig. 5); the data split used in each case is highlighted in the figure.

1. Rendering Model Training: We trained our synthetic image rendering model using pairs of clinical photographs and ground truth HSIs from the training set (Sec. 2.3.2.2).
2. Synthetic Image Generation: We used the trained rendering model to predict a synthetic image for each ground truth HSI (Sec. 2.3.2.3). This provided our paired data necessary for subsequent pipeline training, validation, and evaluation on the test set.
3. Reconstruction Model Training: We used training and validation set synthetic image and ground truth HSI pairs in the reconstruction model training procedure (Sec. 2.4.1). As previously discussed, validation set pairs were used to adjust the learning rate and stop training early if the model was optimized.
4. Test Set HSI Reconstruction: We used the trained reconstruction model to predict an HSI reconstruction for each test set sample (Sec. 2.4.1). Clinical photographs were used as input when they were available for a given sample. Otherwise, prediction was done on synthetic images. This mirrored the way erythema and hyperpigmentation were annotated, which was also done on clinical photographs when available.
5. Test Set Chromophore Abundance Mapping: We used our unmixing method to produce a melanin and hemoglobin abundance map for each test set HSI reconstruction (Sec. 2.4.2).

**Fig. 5.**
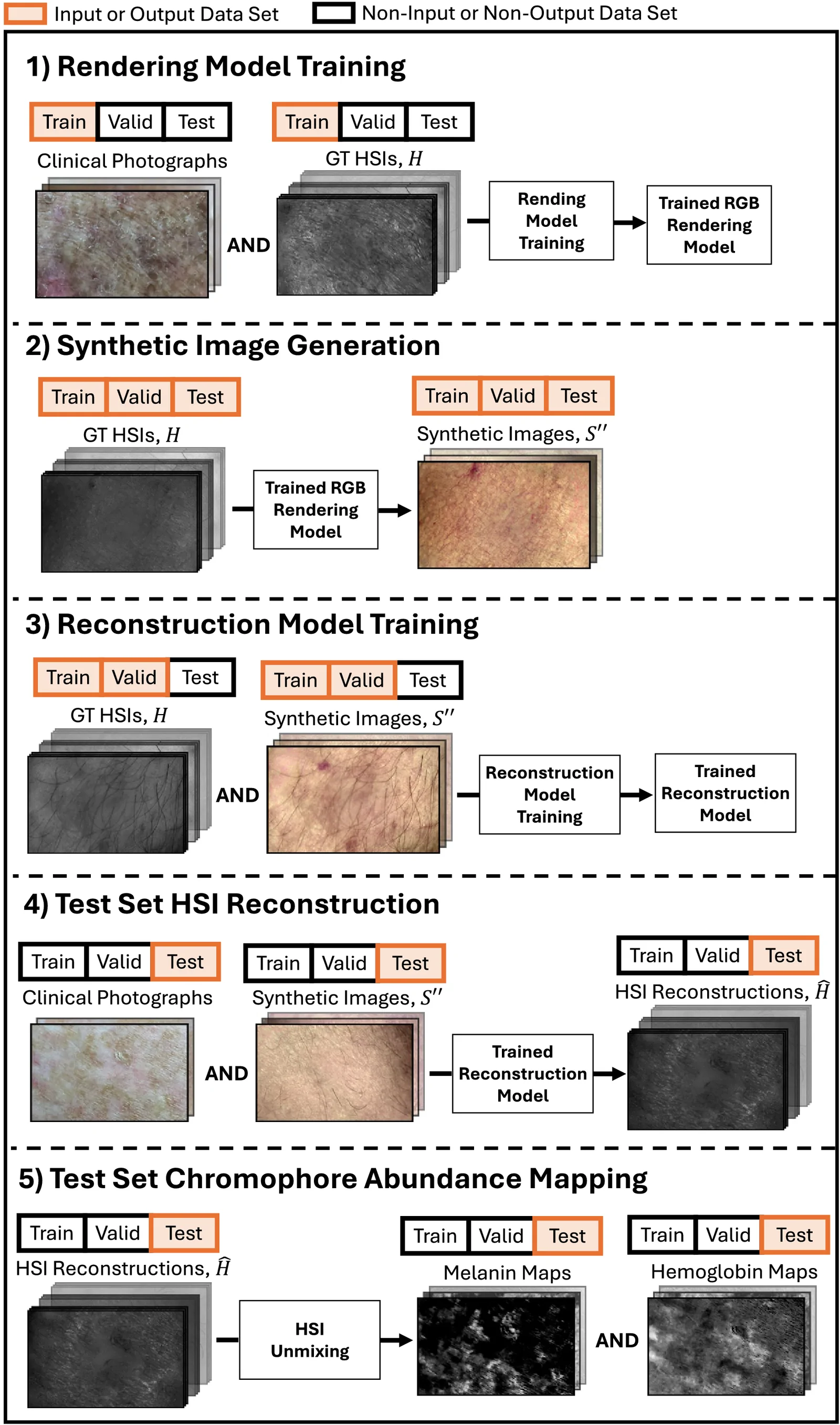
The steps performed for each of our 15 experiments. The data groups (train, test, or valid) used in each step are outlined and shaded in orange, with unused groups outlined in black.

We collected the HSI reconstructions (output of step four) and chromophore abundance maps (output of step five) across our 15 experiments. Only HSI reconstructions derived from synthetic images were used for evaluation, while those predicted on clinical photographs were excluded. Clinical photographs were manually aligned with the corresponding sample’s ground truth HSI, but they were not perfectly registered. Because our per-spectrum validation required comparing each spatial location in a reconstruction directly against the corresponding location in the ground truth HSI, imperfect registration makes this comparison unreliable. Thus, we used 33 HSI reconstructions and 51 hemoglobin and melanin map pairs for our evaluation.

#### 2.5.2. Pipeline evaluation

With these predicted reconstructions and abundance maps, we evaluated the pipeline in two parts: the fidelity of the HSI reconstructions and the agreement of the chromophore abundance maps with expert-approved labels. To evaluate the quality of our HSI reconstructions, we performed a qualitative and quantitative comparison to the corresponding ground truth HSIs. For quantitative validation, we used metrics commonly used to assess spectral recovery models [11]: RMSE and Mean Relative Absolute Error (MRAE). We formulated these metrics on our ground truth test HSI *H* and prediction HSI $\hat{H}$ , pixel set Ω , and HSI channels C=43 , with $\varepsilon =10^{-8}$ for numerical stability. We computed MRAE as

(28) $$\text{MRAE}=\frac{1}{C\;|\Omega |}\overset{\left|\Omega \right|}{\underset{i\in \Omega }{\sum }}\overset{C}{\underset{c=1}{\sum }}\frac{\left|{\hat{H}}_{c,i}-H_{c,i}\right|}{|H_{c,i}|+\varepsilon },$$

and we computed RMSE as

(29) $$\text{RMSE}=\sqrt{\frac{1}{C\;|\Omega |}\overset{\left|\Omega \right|}{\underset{i\in \Omega }{\sum }}\overset{C}{\underset{c=1}{\sum }}{\left({\hat{H}}_{c,i}-H_{c,i}\right)}^{2}},$$

where the sum runs over the same pixel set Ω and the C=43 reconstructed channels.

Figure 6 summarizes the quantitative validation of our abundance maps. To evaluate our chromophore abundance maps for delineating erythema and hyperpigmentation, each map was paired with the corresponding label set for that condition in the same sample (Fig. 6, Step A). Hemoglobin abundance maps were evaluated against erythema label sets, whereas melanin abundance maps were evaluated against hyperpigmentation label sets. If a condition was not present in a given sample, the corresponding abundance map was not used for subsequent analysis. For each condition, DNM pixels were treated as positive labels (indicating regions affected by the condition) and DNI pixels were treated as negative labels (indicating regions unaffected by the condition). Pixels outside of the annotated DNM/DNI regions were excluded from quantitative evaluation, as the annotator was not confident in their classification using the definitely affected and definitely unaffected labelling scheme. These matched label set and abundance map pairs were the common input to both the subsequent receiver operating characteristic (ROC) analysis and the binary classification.

**Fig. 6.**
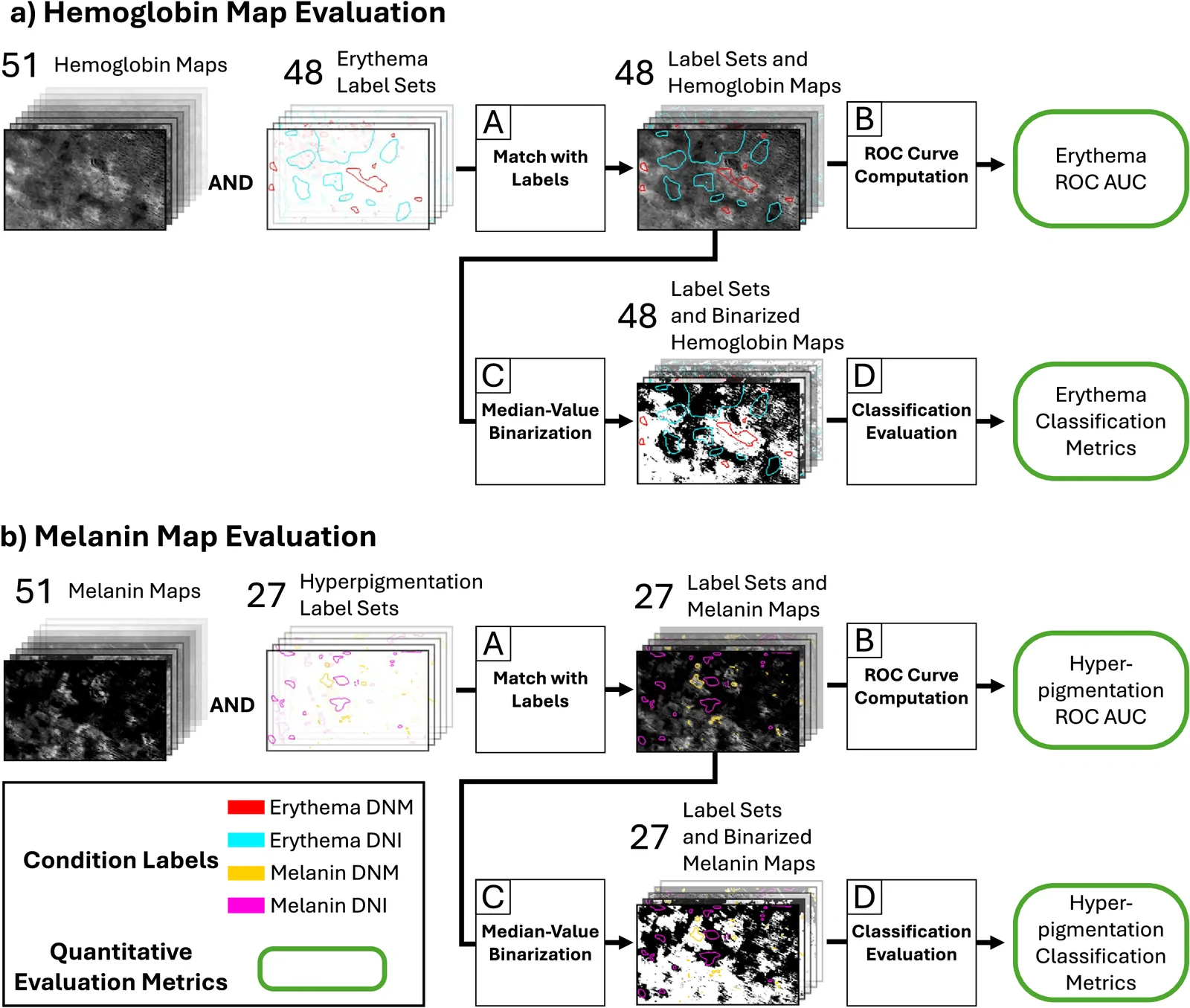
The processing for quantitative validation of our abundance map predictions against DNM and DNI labels. We produced quantitative metrics across both conditions and on synthetic image and clinical photograph input. We assessed ROC area under the curve (ROC AUC) for hemoglobin predicting erythema and melanin predicting hyperpigmentation. Similarly, we assessed erythema and hyperpigmentation classification sensitivity, specificity, and accuracy using binarized abundance maps.

We first assessed the agreement between the continuous abundance values and the expert-approved annotations using ROC analysis (Fig. 6, Step B). For each sample and target condition, the chromophore abundance values within the annotated DNM and DNI regions were used as continuous prediction scores, with DNM pixels assigned the positive class and DNI pixels assigned the negative class. The ROC curve summarizes the tradeoff between sensitivity and false-positive rate over all possible abundance thresholds. The area under the ROC curve (AUC) was used as a measure of separability between annotated positive and negative regions. An AUC close to 1 indicates that abundance values are consistently higher in DNM regions than in DNI regions, reflecting strong alignment between the chromophore abundance map and the labels. An AUC near 0.5 indicates little or no discriminative information, while an AUC below 0.5 would indicate an inverse relationship between abundance and the target label. This analysis yielded the erythema ROC AUC from the hemoglobin maps and the hyperpigmentation ROC AUC from the melanin maps (Fig. 6, green outputs).

The ROC AUC evaluates agreement between our chromophore abundance maps and condition labels but does not assess fixed binary predictions (i.e., using the abundance maps to delineate each condition). To enable this analysis, we also evaluated the performance of binary classification. Traditionally, a threshold could be selected from the ROC curve to optimize agreement with the annotations; however, doing so would make the classification method dependent on human annotations. To avoid annotation-derived threshold selection, each abundance map was binarized using the median abundance value within the map (Fig. 6, Step C). Specifically, pixels with hemoglobin abundance above the sample-specific median were classified as erythema-positive, and pixels with melanin abundance above the sample-specific median were classified as hyperpigmentation-positive. Pixels at or below the given map’s median abundance were classified as condition-negative. We evaluated these predictions against the label sets, and computed the resulting sensitivity, specificity, and accuracy for each condition across our samples (Fig. 6, Step D).

Thus, our analysis quantified not only whether the continuous abundance maps were aligned with expert-approved annotations, but also whether a simple annotation-independent threshold produced condition classifications consistent with the DNM/DNI labels.

## 3. Results and discussion

We present our results in two parts, corresponding to the two stages of the pipeline. First, Section 3.1 evaluates the reconstruction model by comparing the predicted HSI reconstructions to ground truth HSIs. Second, Section 3.2 evaluates the proposed HSI unmixing method by assessing the chromophore abundance maps against expert-approved erythema and hyperpigmentation annotations.

### 3.1. Evaluation of HSI reconstruction

Figure 7 provides a qualitative summary of our results for three representative samples—one displaying erythema, one hyperpigmentation, and one with both conditions—at each stage of our pipeline: synthetic image input, ground truth (GT) and reconstructed (Pred) HSI bands, and the subsequent hemoglobin and melanin abundance maps. The representative samples were chosen because they have regions with large labeled areas located away from image corners, where imager-setup artifacts were more common. We provide the RMSE of each predicted HSI reconstruction patch underneath the representative HSI band images in the middle column. The hemoglobin and melanin abundance maps in the third column highlight representative erythematous (indicated by red arrows) and hyperpigmented (indicated by brown arrows) regions, respectively.

**Fig. 7.**
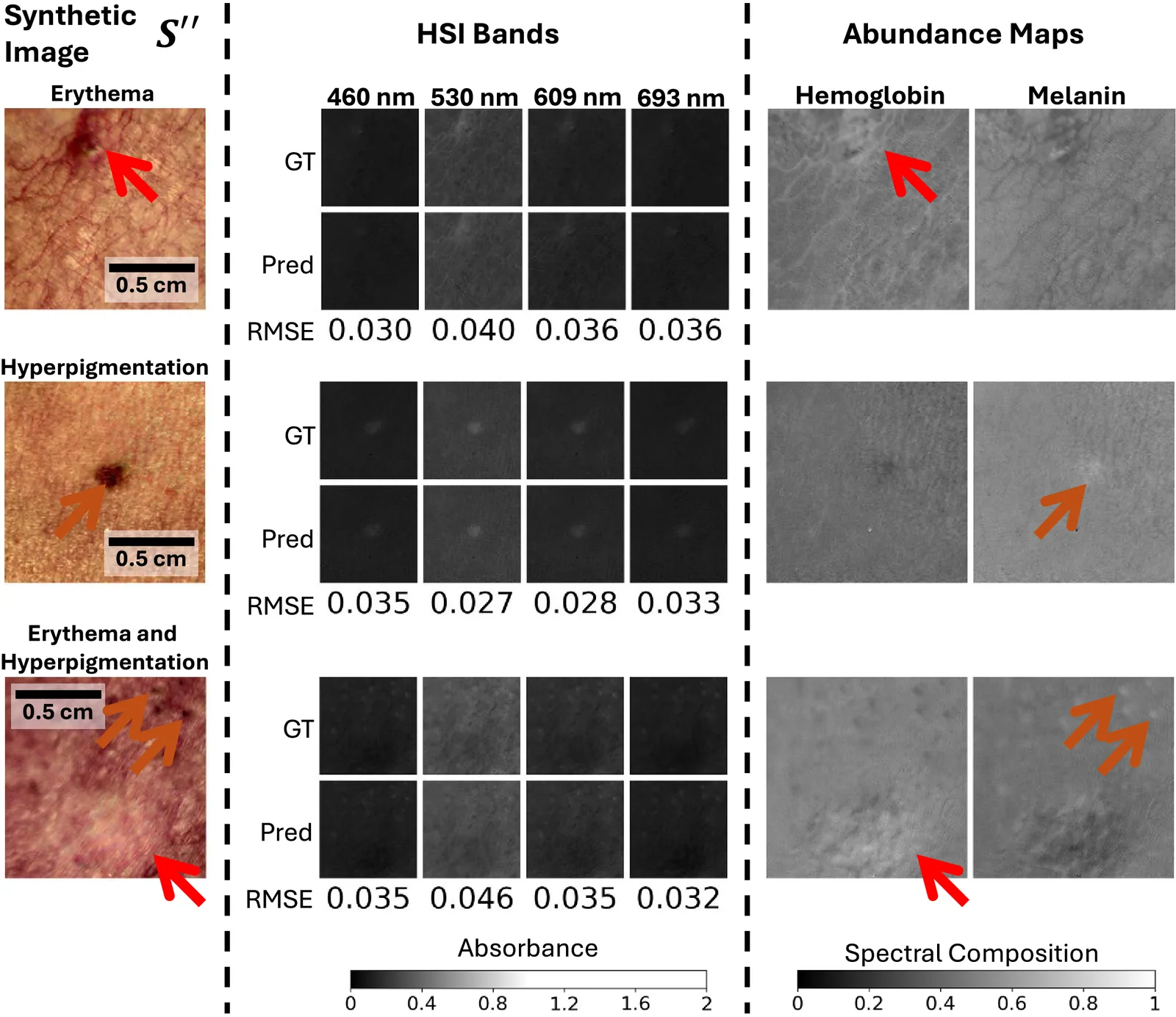
Qualitative summary of our approach for samples displaying erythema, hyperpigmentation, and both, as indicated by expert-approved labels. HSI bands were chosen to span the entire reconstructed wavelength range, and reconstruction RMSE is displayed for each band. Hemoglobin maps predict erythema and melanin maps predict hyperpigmentation. Arrows mark representative labeled affected regions (red for erythema, brown for hyperpigmentation). Each patch displays a 1.67×1.67 cm region.

Since we estimate HSIs in absorbance units that were clamped in the range [0,2] as a result of conversion from reflectance units, an RMSE of 0.1 corresponds to 5% of the target data range. Notably, we achieve an RMSE below this value for each HSI patch seen in Fig. 7. Furthermore, across our predicted HSIs, 95.98% of the wavelength bands displayed an RMSE below 0.1. Thus, we conclude that we obtained accurate reconstruction of HSI bands across the wavelength range. Importantly, the chromophore abundance maps show delineation of erythema via hemoglobin abundance and hyperpigmentation via melanin abundance. The results visually make sense: areas appearing red in the synthetic images that evoke a sense of blood-rich regions map to regions of higher hemoglobin abundance, and brownish skin areas map to more melanin-rich regions.

Table 2 summarizes this quantitative analysis of our reconstructions in terms of mean metric values and standard deviations across our samples. Our quantitative results were comparable to the state-of-the-art MST++ performance on the benchmark ARAD1 K spectral recovery dataset [11], suggesting the proposed architecture produces accurate hyperspectral reconstructions from our cutaneous synthetic images. Specifically, an MRAE of 0.09 across our dataset suggests that absorbance predictions had a low error relative to their true values.

**Table 2.** Quantitative evaluation of HSI reconstruction (metric mean with standard deviation) compared to benchmark performance on the ARAD1 K dataset^*a*^

**MST++ Spectral Recovery Performance**		
	Synthetic Image Dataset	ARAD1 K Benchmark

RMSE	0.05 ± 0.03	0.02
MRAE	0.09 ± 0.04	0.16

^*a*^ Lower metric values indicate higher-quality reconstruction.

### 3.2. Evaluation of chromophore abundance mapping

We evaluated our chromophore abundance maps in two ways: first, using a threshold-independent analysis of how well the continuous abundance values separate labeled regions, and second, via an annotation-independent binary classification for each condition.

Figure 8 displays the per-sample and average (in bold) ROC curves for both synthetic image and clinical photograph inputs. We achieved a mean ROC AUC > 0.85 for both erythema and hyperpigmentation labels, indicating that our reconstruction and unmixing approach demonstrates strong predictive capability for these conditions. Additionally, we demonstrate strong performance on both synthetic images and clinical photographs. This demonstrates that the HSI reconstruction model is able to generalize to true photographs despite being trained only on our synthetic images. Furthermore, our subsequent abundance mapping method performs well on HSI reconstructions derived from both synthetic images and photographs.

**Fig. 8.**
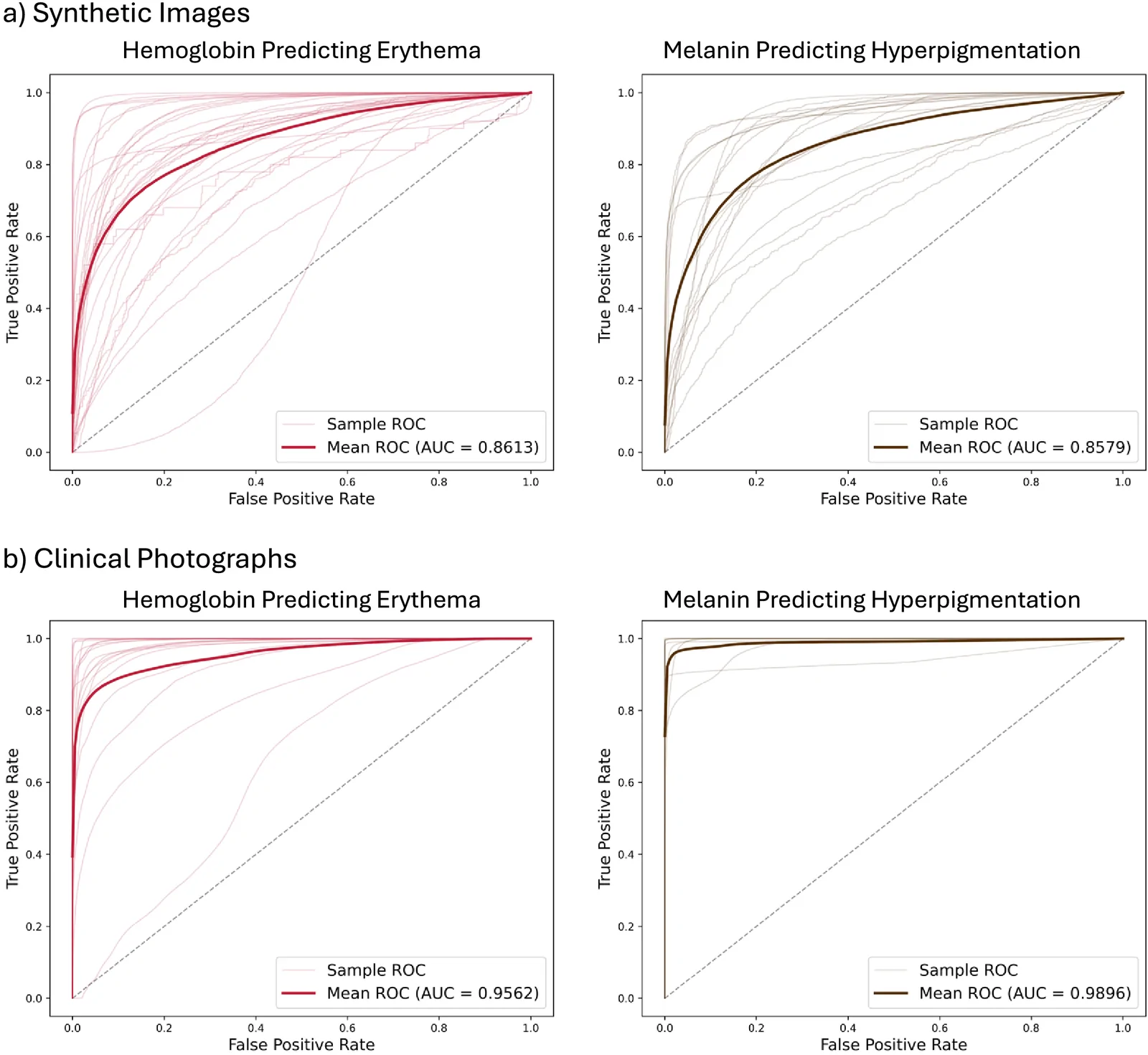
Per-sample and mean ROC curves for abundance maps on expert-approved annotations. Higher area under the curve (AUC) indicates stronger separation of chromophore abundance between DNI and DNM regions.

Figure 9 displays the per-sample distribution of each of our quantitative evaluation metrics across both synthetic images and clinical photographs. The ROC AUC panel summarizes the per-sample values underlying the curves in Fig. 8. For hemoglobin predicting erythema (Fig. 9(a)), hemoglobin abundance separated the labeled regions strongly, with a median ROC AUC of 0.89 on synthetic images and 0.99 on clinical photographs. For classification, median sensitivity and specificity were both 0.80 or greater on both modalities, and median accuracy improved from 0.72 on synthetic images to 0.82 on clinical photographs. Clinical photographs improved on synthetic images for ROC AUC, sensitivity, and accuracy, while specificity was comparable between the two.

**Fig. 9.**
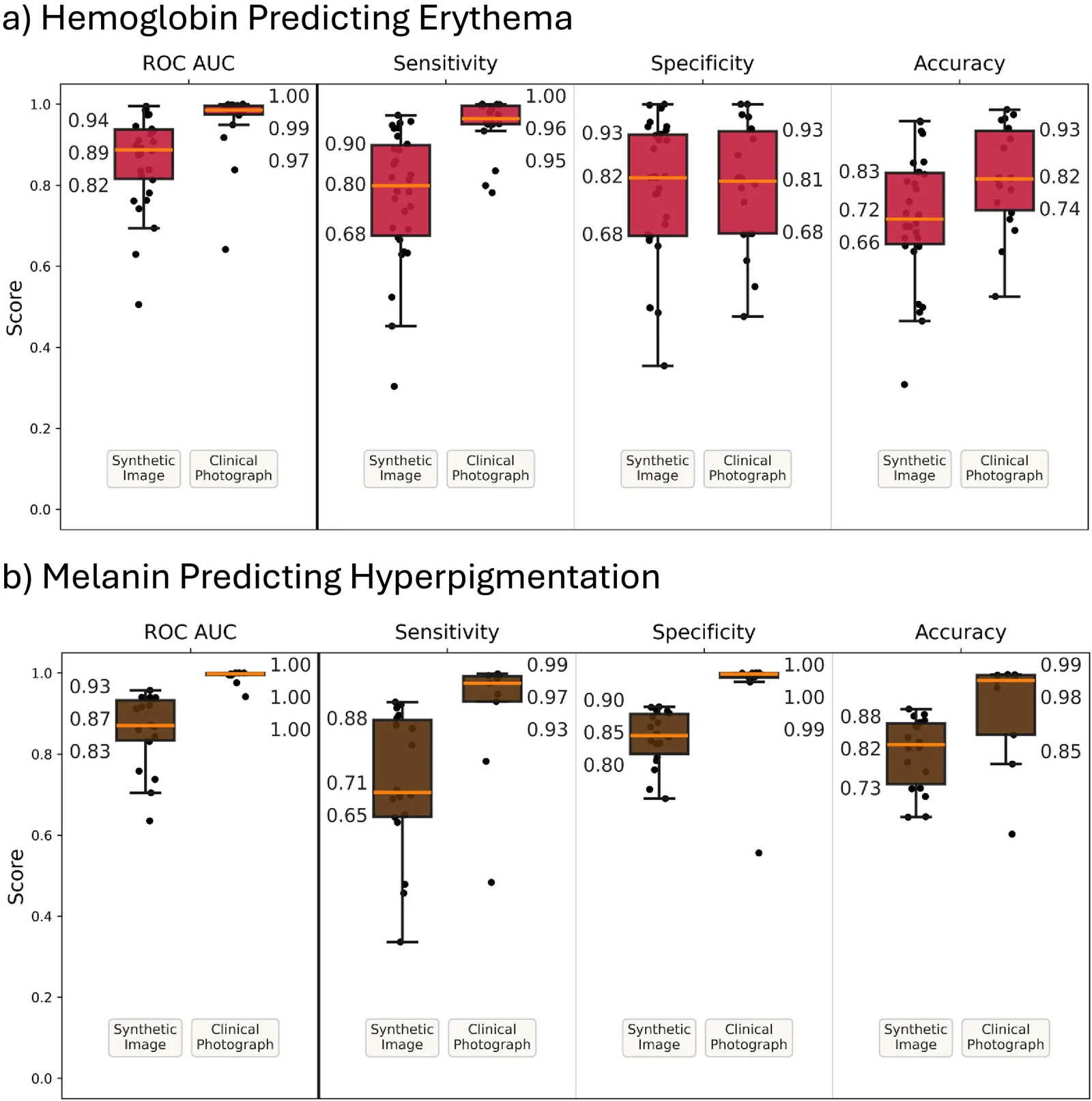
Box plots summarizing classification performance with dots representing individual sample scores. The ROC AUC panel summarizes the per-sample AUCs underlying the curves in Fig. 8 and is computed on the continuous abundance values (before binarization), while sensitivity, specificity, and accuracy use median-value binarization of each map. The median, 25^th^ and 75^th^ percentiles are labeled for each sample group.

For melanin predicting hyperpigmentation (Fig. 9(b)), median ROC AUC was again high, at 0.87 on synthetic images and 1.00 on clinical photographs. Median specificity was strong on both modalities (0.85 on synthetic images and 1.00 on clinical photographs), while median sensitivity was 0.71 on synthetic images and 0.97 on clinical photographs, and median accuracy rose from 0.82 to 0.98. In contrast to erythema, clinical photographs exceeded synthetic images on all four metrics for hyperpigmentation.

Our worst performing median classification metric across both modalities and conditions was a hyperpigmentation sensitivity of 0.71 on synthetic images. This indicates that 71% of definitely affected (DNM) pixels were correctly predicted under a purely annotation-independent median threshold, which is still well above the 0.5 chance level, and it improves substantially (to 0.97) on clinical photographs.

Overall, our results indicate strong erythema and hyperpigmentation classification when evaluated against DNM and DNI labels. Our quantitative results further suggest that our methods generalize from synthetic images to true photographs. Notably, the classification maps were generated by binarizing the abundance maps at their median values. Our ROC analysis, with a median AUC of 0.87 or higher across both conditions and image modalities, suggests that classification performance could improve further with thresholds calibrated on a held-out sample set. Importantly, our method does not rely on human annotations, and thus, the provided results indicate strong agreement between our independent physics-guided approach and the expert understanding of erythema and hyperpigmentation.

These results can also be compared to the previously published analysis of this same cohort performed directly on the measured hyperspectral data [10], which reported median segmentation accuracies of 0.83 for erythema and 0.73 for hyperpigmentation using supervised chromophore mapping. Our RGB-derived delineations achieve comparable agreement with the same expert-approved annotations (median accuracy 0.72 on synthetic images and 0.82 on clinical photographs for erythema; 0.82 and 0.98 for hyperpigmentation) despite requiring no hyperspectral imager at the point of care. Furthermore, our annotation-independent abundance map binarization method contrasts with the work presented by Saknite et al., which selected thresholds to maximize median accuracy across the annotation set.

## 4. Conclusion

We have demonstrated a method for erythema and hyperpigmentation delineation for cutaneous cGVHD on RGB images (i.e., those captured from a standard digital camera) that does not rely on human annotations to produce estimations. Despite not using them to inform our approach, our results suggest that the predictions produced by our approach show high agreement with high-confidence annotations. Although we trained our reconstruction model on only synthetic RGB images, we demonstrated that our methods generalize to true clinical photographs of cutaneous cGVHD. Our physics-guided approach enables objective and reproducible delineations that are informed by cutaneous chromophores rather than the conventions of specific annotators. Because erythema and hyperpigmentation predictions are produced from RGB images, the presented work is significantly cheaper and easier to incorporate into the clinical workflow than alternative approaches for producing objective delineations (i.e., using a hyperspectral imager in the clinic).

Our evaluation was based on a modest dataset of 51 samples from 15 patients. Considering the similarity to data sizes collected in other published work [12,30–32], we consider this data sufficient to support the claims made here, and our presented results are consistent with a previously published analysis of the same cohort performed directly on measured hyperspectral data [10]. The present results should be interpreted as establishing feasibility rather than full clinical validation. While we demonstrated strong agreement with expert-approved annotations for both erythema and hyperpigmentation across our dataset, the imaged region in each sample was only 2.5×3.5 cm. Thus, we are unable to determine the ability of our method to estimate full erythema BSA for a given patient. A dataset that captures a larger BSA for each patient—either wide-field HSIs or a sufficient number of narrow-FOV HSIs sampled across the body—would be necessary to enable this analysis. Furthermore, our dataset contained only one patient with a dark skin tone, with two corresponding samples. While erythema was not present in either sample, our method achieved a mean sensitivity, specificity, and accuracy of 0.86, 0.81, and 0.85, respectively, for hyperpigmentation prediction for this patient. A larger dataset with more diverse skin tones would be needed to evaluate if our methods provide suitable delineations for patients across the range of skin tones. The patients’ Fitzpatrick skin type was not available for our analysis. This factor should be recorded in future datasets, and accounting for it—for example, in the reference endmember set—could improve performance across skin tones.

The presented pipeline reconstructs and unmixes 43 hyperspectral bands, and we did not optimize the number or wavelengths of these bands. A considerably smaller, task-optimized set of wavelengths may enable comparable accuracy for hemoglobin and melanin abundance estimation. Establishing such a minimal band set would reduce the cost of the hyperspectral measurements needed to supervise the reconstruction stage, which is the principal barrier to assembling a larger and more skin tone diverse dataset.

Similarly, because the available annotations were constrained to high-confidence regions, across our label sets, very few regions were labeled DNM for both erythema and hyperpigmentation (0.002% of pixels across all samples). Thus, we are unable to adequately investigate the accuracy of methods when predicting this clinically relevant scenario (i.e., co-occurring erythema and hyperpigmentation in a given cutaneous region).

Additionally, because the hyperspectral measurements analyzed here were acquired with a standardized acquisition procedure with a fixed illuminant and posing, our results on synthetic images do not characterize robustness to the illumination and camera variability that would be encountered in routine clinical care. Our results on the 18 clinical photographs, which were not collected under the same acquisition procedure, demonstrate generalizability beyond these synthetic images, but similarly do not demonstrate robustness to a range of collection conditions. Prospective acquisition across multiple cameras, lighting conditions, and a wider range of skin tones is a necessary step toward deployment.

The presented work provides a path to more consistent and accessible assessment for cutaneous cGVHD progression, which could enable improved care and improved patient outcomes. Furthermore, because erythema BSA is used as a criterion for assessing drug efficacy in clinical trials, more objective and reproducible erythema delineation could enable improved drug development.

## Data Availability

The code, model weights, and data that support the findings of this study are available from the corresponding author upon reasonable request.
